# Practical Chemistry and Pharmacy

**Published:** 1894-04

**Authors:** 


### PRACTICAL CHEMISTRY AND PHARMACY.

EDITED BY HENRY R. SLACK, M. D., PH. M.

### ANTIDOTE TO MORPHINE.

Dr. Wm. Moore, of New York City, has proposed permanganate of potash as an antidote to morphine, and that he has faith in the proposition is shown by the fact that he has tried the experiment on himself by taking toxic doses of the salt and then using the potassium permanganate as an antidote, with the happiest results. It has been doubted whether the permanganate would counteract the effect of the morphine after it had been absorbed. Further experiments, made on animals, have shown that, with them at least, the poison may be neutralized after it has entered the circulation, by applying the antidote as a hypodermic injection, and following these a case is reported in which the treatment was successful in a human subject.

The experiments on rabbits are interesting. Five subjects were taken. In the first one grain was given by the stomach. No antidote was used, and the rabbit died in three hours. The second rabbit was given a grain of the poison hypodermically, and fifteen minutes after, three-fourths of a grain permanganate in the same way, followed by one-third of a grain fifteen minutes later. In two hours it could move, though sluggish. The third rabbit was given two grains of morphine through the mouth at 4:52 P. M. Respiration had dropped to 14 at 5:30, and at 6:45 to 12. An intra-venous injection of two grains of the permanganate was then administered, with the result that in ten minutes respiration had risen to 26. It fell again, however, to 20, but became stronger after the lapse of half an hour, when it registered 34. The animal was then pronounced to be out of danger, and after an interval of an hour, was apparently as well as ever.

The fourth rabbit received an intra-venous injection of one grain of the poison, and exhibited almost exactly the same symptoms as those observed in rabbit No. 1, who received the same quantity through the mouth.

The fifth rabbit received five grains morphine sulphate through the mouth at 5:48, and at 7 o'clock was sinking rapidly, when he was treated with a solution of six grains of potassium permanganate, injected through the veins. Within half an hour he began to show signs of improvement, and by 8 o'clock had almost entirely recovered from the effects of the poison.

According to press dispatch, this experiment has been tried on a human subject at Paragould, Ark. A man suffering from despondency took three grains of the poison, and after his pulse had fallen to 40 and respiration to 10, was saved by hypodermic injection of the permanganate.

Dr. Moore suggests that in case of the poisoning by the alkaloid or tincture of opium, some diluted sulphuric acid or vinegar should be given with the antidote, to convert insoluble morphia into a more soluble salt.—*Druggist Circular.*

---

### CREOSOTE PILLS.

E. C. Goetting (*Pharm. Rundschan,* 1894, xii., p. 34) states to have found, after numerous experiments, the following recipe to be excellent: To prepare 100 pills, each containing 10 centigrammes (1½ min.) of creosote, 5 grammes (77 gr.) of benzoin, all reduced to a fine powder in a pill mortar (the powdered benzoin of the market being unfit for the purpose), and 10 grammes (2½ drs.) of creosote are thoroughly triturated with the powder, until the benzoin is dissolved. To this solution are added 2½ grammes (38 grs.) of finely pulverized borax and 20 drops of glycerine, and the mass is triturated with 13–15 grammes (3¼–3¾ drs.) of powdered licorice root to form a soft pill-mass, which is at once made into 100 pills. The mass is easily rolled out. These pills leave no greasy spot when crushed on paper, do not flatten while lying, soon harden into a durably plastic consistence, and easily dissolve in water, even after having been kept several weeks, it is claimed. The emulsification of the creosote is so complete that the pills, in being rubbed up with water, give a uniform emulsion, depositing only the licorice root, and which may well serve as creosote emulsion.

[Having tried this formula we can recommend it as making a splendid pill.—H. R. S.]

---

### PRESERVATION OF DILUTE SUBLIMATE SOLUTION.

As is known 1:1000 sublimate solutions are not employed on a large scale for surgical antisepsis. When prepared simply with distilled water, L. Vignon (*Rép de Pharm.,* 1894, VI., p. 5) states that after one to three days, a slight white precipitate forms, which increases with time. A liter of this solution, kept uncovered in the laboratory for seven days, at a temperature varying between 15° to 20° C. (59 to 68 F.), contained only 57 centigrammes of corrosive sublimate. The same quantity of a similar solution, prepared in glass stoppered bottle, contained 97 centigrammes of sublimate after seven days, and 67 at the end of two hundred and twenty days. The author states that fuchsine and indigo-carmine, frequently used for coloring sublimate solutions, diminish the precipitation, indigo-carmine being more active in this regard than fuchsin; and that if to a liter (33.8 fl. oz.) of the sublimate solutions there be added one gramme (11 min.) of hydrochloric acid, or 10 grammes (2½ drs.) of sodium or potassium chloride, they retain their limpidity for a much longer time. Tauret (*ibid.*) attributes the deterioration to the influence of atmospheric ammonia.—*Merck's M. R. and Phar. Journal.*

---

### DISCOLORIZATION OF IODINE IN LINIMENTS.

The discolorization noted when iodine is brought into contact with certain essential oils is due in some cases at least to the formation of hydriotic acid, hydrogen being abstracted from the oil, and this acid being colorless, the original tint of the mixture of course disappears. Possibly other colorless iodine products may be found at the same time, or in the case of some oils. The subject does not seem to have been thoroughly studied.

It is manifest that when the reactions above noted have occurred, both the iodine and the essential oil have been greatly changed, and the prescriber or formula-maker has something very different from what he set out to make. Whether the therapeutic effect would be changed, or to what extent, could not be determined by clinical experiment.—*Drug Circular and Chemical Gazette.*

---

### FERRATIN.

This is the name given to a substance isolated from pig's liver by Schmiedeberg. It contains, on the average, six per cent. of iron, and is regarded as the organic iron compound nominally present in the organs of the animal body, being ingested with food and stored up in the tissues as a reserve material for the formation of blood. The natural ferratin being difficult to obtain in quantity, the compound is prepared artificially by treating egg albumin, dissolved in water, with the tartrates of potassium and iron, together with soda lye. The resulting precipitate is described as a reddish-brown, inodorous, and tasteless powder, of neutral reaction, and containing seven per cent. of readily assimilable iron. A sodium compound of ferratin is also prepared, which is soluble in water. The dose of either form is 1 to 1.5 grains divided into two or three portions.—*Drug Circular and Chemical Gazette.*

